# Exosomal miR-1305 in the oncogenic activity of hypoxic multiple myeloma cells: a biomarker for predicting prognosis

**DOI:** 10.7150/jca.55553

**Published:** 2021-03-14

**Authors:** Ji Young Lee, Daeun Ryu, Sung Won Lim, Kyung Ju Ryu, Myung Eun Choi, Sang Eun Yoon, Kihyun Kim, Chaehwa Park, Seok Jin Kim

**Affiliations:** 1 Department of Health Sciences and Technology, Samsung Advanced Institute for Health Sciences and Technology, Sungkyunkwan University, Seoul, Korea.; 2 Department of Medical Informatics, College of Medicine, The Catholic University of Korea, Seoul, Republic of Korea.; 3 Division of Hematology-Oncology, Department of Medicine, H plus Yangji hospital, Seoul, Korea.; 4 Division of Hematology-Oncology, Department of Medicine, Samsung Medical Center, Sungkyunkwan University School of Medicine, Seoul, Korea.

**Keywords:** exosome, microRNA, miR-1305, multiple myeloma, hypoxia

## Abstract

**Background:** Exosomes have emerged as important mediators of tumor progression, and a prognostic role for serum exosomal miRNAs has been suggested in multiple myeloma (MM). Given the association of hypoxia with tumor aggressiveness, including cancer stem cell-like phenotypes, we explored exosomal miRNAs from MM cells under hypoxic conditions and analyzed their diverse roles both in promoting oncogenic activity and in predicting prognosis.

**Methods:** The human MM cell line, RPMI 8226, was cultured under hypoxic conditions and their exosome production and exosomal miRNA profiles were compared with those of normoxic parental cells. The survival outcome of myeloma patients was compared using serum levels of exosomal miRNAs, and the effects of exosomal miRNAs on the target genes of MM cells and adjacent immune cells were analyzed.

**Results:** Increased expression of stem cell markers and exosome production were observed in hypoxic MM cells. Exosome miRNA analysis identified a higher expression of miR-1305 in exosomes isolated from hypoxic MM cells than in those of normoxic parental cells. The overall survival of patients with high exosomal miR-1305 was poorer than it was in patients with low exosomal miR-1305. In hypoxic MM cells, an increase of exosomal miR-1305 led to a decrease of cellular miR-1305 and increased expression of the miR-1305 target genes, *MDM2, IGF1* and *FGF2* resulted in the promotion of oncogenic activity of MM. Exosomal miR-1305 was also transferred from MM cells to macrophages, and miR-1305-transferred macrophages showed tumor-promoting, M2-macrophage phenotypes.

**Conclusions:** Exosome-mediated secretion of miR-1305 in MM cells promoted oncogenic activity of hypoxic MM cells and high serum levels of exosomal miR-1305.

## Introduction

Multiple myeloma (MM) is the second most common hematologic malignancy and is characterized by the clonal proliferation of malignant plasma cells in bone marrow (BM) 1, 2. Despite significant advances in the treatment of MM, including proteasome inhibitors, immunomodulatory drugs and monoclonal antibodies, MM remains incurable because most patients experience recurrent relapse 3-7. The high frequency of relapse in MM is known to be related to the heterogeneity of tumor cells. Thus, the more aggressive subsets of cells resistant to drugs - the so-called myeloma stem cells - could exist in the BM microenvironment resulting in treatment failure in MM patients 8, 9. Although the underlying mechanisms of cancer stem cell development are still poorly understood, the acquisition of stem cell-like phenotypes is strongly associated with the adaptation of tumor cells to hypoxic conditions 10, 11. Indeed, hypoxia in the tumor microenvironment is a well-known factor contributing to tumor progression and drug resistance 12, 13. As the oxygen pressure in BM is lower than that in peripheral blood, the association of hypoxia with a stem cell-like phenotype and with the drug resistance of MM cells has been reported 14, 15.

Exosomes of 30-150 nm are endosome-derived extracellular vesicles termed 'small extracellular vesicles'; they are cell-derived, small, membrane-bound particles secreted from various types of cells including tumor cells 16-18. Tumor cells are able to release exosomes that promote their growth and metastasis, because exosome cargo includes proteins, lipids, DNAs, messenger RNAs and microRNAs (miRNAs) 19, 20. In particular, small noncoding RNAs are enriched in exosomes, and exosomal miRNA has been suggested as a biomarker that reflects tumor aggressiveness and patient outcomes 21, 22. Various miRNA abnormalities have been reported in myeloma patients, suggesting miRNAs play a role in the pathogenesis of MM 23. For example, expression of the tumor suppressors miR-15a and miR-16 is markedly reduced in MM due to deletion of chromosome 13q14, preventing their ability to inhibit cell proliferation 24. In contrast, oncogenic miRNAs such as miR-21 and miR-221 are highly expressed, resulting in a decrease of apoptosis and the growth of MM cells 25, 26. The expression of exosomal miRNA also shows an association with the expression levels of myeloma-related factors including β2-microglobulin and IL-6 27. Given that exosomal miRNAs can be obtained from peripheral blood, unlike conventional genomic approaches that require a bone marrow biopsy, they could be candidates for use as noninvasive biomarkers in MM. Therefore, we explored exosomal miRNAs from MM cells under hypoxic conditions and analyzed their association with the aggressiveness of MM cells and prognosis in MM patients.

## Materials and Methods

### Cell culture

Human cell lines, RPMI 8226 and THP-1, were purchased from the ATCC (Rockville, MD). Human myeloma RPMI 8226 cells were grown in RPMI 1640 culture medium supplemented with 10% heat-inactivated fetal bovine serum (FBS), penicillin and streptomycin (Gibco-BRL, Grand Island, NY). Human monocytic THP-1 cells were cultured in RPMI 1640 medium containing 10% heat inactivated FBS and supplemented with 10 mM HEPES, 1 mM pyruvate, 2.5 g/L D-glucose, penicillin, streptomycin (Gibco-BRL) and 50 pM β-mercaptoethanol (Gibco-BRL).

### Hypoxic culture and exosome isolation

Exosomes were isolated from culture medium using ExoQuick-TC precipitation solution (System Biosciences, Mountain View, CA) according to the manufacturer's instructions as we isolated exosomes from lymphoma cell lines in our previous study 28. RPMI 8226 cells were cultured for 2 to 4 weeks under hypoxic (2% O_2_ and 1% O_2_) or normoxic (21% O_2_) conditions and then culture medium was replaced with 10% exosome-depleted FBS (Gibco) for 72 h. Culture medium was collected and differentially centrifuged at 300 g for 10 min, 2000 g for 10 min and 10,000 g at 4 °C for 30 min and then passed through a 0.22-μm filter to remove cell debris. Clarified cell culture medium was mixed with ExoQuick-TC solution and incubated at 4 °C overnight before centrifugation twice at 1,500 g for 30 min and 5 min, respectively, in order to remove the supernatant. The pellet was resuspended in 100 μL of phosphate-buffered saline (PBS).

### Exosome isolation from serum samples of myeloma patients

The study population was selected from myeloma patients enrolled into our previous two cohort studies that were approved by the institutional review board at the Samsung Medical Center (IRB number 2009-08-065 and 2013-09-009). As the cohort study collected serum samples after written informed consent, myeloma patients' serum-derived exosomes could be analyzed for the validation of *in vitro* cell-line study results. Thus, we selected myeloma patients according to the following inclusion criteria. First, patients should have at least 1 mL of serum samples that had never been thawed until analysis. Second, patients should have long-term follow-up data for survival analysis. Third, patients should be initially treated with bortezomib- or thalidomide-containing regimens to minimize the heterogeneity of induction treatment. Accordingly, we selected 42 patients who were initially treated with bortezomib- or thalidomide-containing regimens between 2010 and 2017. The last update of survival and disease status was performed in March, 2020. For the isolation of exosomes, serum was differentially centrifuged at 2,000 g at 4 °C for 10 min and 10,000 g at 4 °C for 30 min, and passed through a 0.22-μm filter to remove cell debris. Clarified serum was mixed with ExoQuick (System Biosciences, Palo Alto, CA) and incubated at 4 °C for 30 min then centrifuged twice at 1,500 g for 30 min and 5 min, respectively to remove the supernatant. The pellet was resuspended in 200 μL of PBS. Our protocol of isolating exosomes from archived patients' serum samples was same as that of our previous study for lymphoma patients 28.

### Transmission electron microscopy (TEM) analysis

Exosomes were fixed in 4% paraformaldehyde and transferred onto Formvar-carbon-coated TEM grids. Fixed samples were allowed to absorb filter-paper for 20 min in a dry environment. Grids were rinsed in PBS. After removing the supernatant liquid by filter paper absorption, grids were floated on a drop of 2.5% w/v glutaraldehyde for 5 min. Grids were washed 10 times with distilled water and negative stained with 1% uranyl acetate for 1 min and then air dried. Grids were observed with a Hitachi TEM 7700 (Hitachi Inc., Dallas, TX) at 80 kV.

### Nanoparticle tracking analysis (NTA) of exosomes

Exosome suspensions with concentrations between 1×10^7^/mL and 1×10^9^/mL were examined using a NanoSight NS300 (NanoSight Ltd., Amesbury, UK) particle analyzer and analyzed using NTA software (version 2.3; NanoSight Ltd.) for the size and quantity of particles isolated.

### RNA extraction and real-time PCR

Total RNA was isolated from cells and exosomes using the miRNeasy Micro kit according to the manufacturer's instructions (Qiagen Inc., Valencia, CA). RNA concentration was measured using the NanoDrop ND-100 Spectrophotometer (NanoDrop Technologies, Wilmington, DE). For miRNA expression analysis, 50 ng of RNA was mixed with TaqMan MicroRNA Reverse Transcription Kit reagent containing specific miRNA primers and reverse transcribed according to the manufacturer's instructions (Thermo Scientific, Rockford, IL). Real-time PCR was performed by diluting the complementary cDNA product in 2× TaqMan Universal Master Mix II and 20× TaqMan microRNA Expression Assay for each mature miRNA to be measured: miR-1305 and miR-21 (Applied Biosystems, Waltham, MA). U6 and cel-miR-39 were used as the normalization controls for cells and extracellular vesicles, respectively, in miRNA expression-level analysis. For quantification of gene expression, 2 μg RNA was converted to cDNA using an Omniscript RT kit (Qiagen, Valencia, CA). The cDNA generated was amplified using TaqMan assay for HIF-1α, Nanog, OCT4, SOX2 and β-actin. Reactions were carried out in a QuantStudio 6 system (Applied Biosystems) and the results expressed as fold change calculated with the comparative CT (ΔΔCt) method relative to the control sample. β-Actin was used as the internal control for mRNA analysis.

### RNA sequencing and data analysis

Whole transcriptome sequencing libraries were constructed using a TruSeq RNA sample preparation v2 kit (Illumina). Sequencing was performed using the 100-bp paired-end mode of the TruSeq Rapid PE Cluster kit and TruSeq Rapid SBS kit (Illumina). The sequencing reads were mapped to the GRCh37.75 human reference genome by using STAR version 2.4.0 26. To quantify gene expression, mapped reads were processed by RSEM version 1.2.18 29. The 'edgeR' method was used to identify differentially expressed genes (DEGs) and the results were filtered on the absolute value of the log 2 (fold change) to more than two 30. We performed gene ontology (GO) analysis using DAVID 31.

### Western blot

Exosomes were lysed in RIPA buffer (0.5% sodium deoxycholate, 1% Nonidet P-40, 150 mM NaCl, 50 mM Tris [pH 7.5], 0.1% sodium dodecyl sulfate [SDS] and 1 mM phenylmethylsulfonyl fluoride [PMSF]) and cleared by microcentrifugation (13,000 rpm for 10 min at 4 °C). In total, 50 μg protein samples were electrophoresed on a 4%-12% SDS polyacrylamide gel (SDS-PAGE) and transferred to nitrocellulose membranes. The membrane was incubated with blocking solution (5% non-fat milk) for 1 h and incubated overnight at 4 °C with primary antibodies including anti-Alix, anti-CD63 and β-actin (Santa Cruz Biotechnology, CA), and HIF-1α, Akt, p-Akt, p-P65, p-ERK, BCL2 and P21 (Cell Signaling, Danvers, MA). The blot was washed with TBST buffer (50 mM Tris [pH 7.5], 150 mM NaCl, 0.05% Tween 20) and further exposed to horseradish peroxidase-conjugated secondary antibody for 1 h at room temperature. Proteins were visualized using enhanced chemiluminescence (ECL) reagent (Invitrogen, Carlsbad, CA).

### NanoString nCounter analyses

For each sample analyzed, total exosomal RNA was used for miRNA profiling by a NanoString nCounter^®^ microRNA platform (version 3) as per the manufacturer's instructions. nCounter miRNA expression assay count data were normalized. Technical normalization was performed according to guidelines provided by NanoString. The nCounter results were analyzed using nSolver 4.0 software according to the manufacturer's instructions.

### MicroRNA transfection

RPMI 8226 cells were transfected with miR-1305_mimic and negative control miRNA (Exiqon, Copenhagen, Denmark) for 48 h and 72 h using Lipofectamine™ RNAiMAX (Invitrogen: Thermo Fisher Scientific, Inc.).

### Transwell coculture assay

THP-1 monocytes (3×10^5^) cells were seeded into a 24-well plate (lower chamber) and differentiated with 150 nM phorbol 12-myristate 13-acetate (PMA, Sigma-Aldrich, St. Louis, MO) for 24 h, after which the PMA-containing medium was replaced with 10% FBS-containing 1× RPMI growth medium for 24 h. Then, RPMI 8226 transfected cells were plated in a Transwell chamber (upper chamber pore size, 0.4 μm; Corning Costar, Tewksbury, MA). This cell density was chosen in order to have a 1:1 ratio between macrophages and RPMI 8226 cells cocultured in 10% exosome-depleted FBS culture medium.

### Statistics

The correlations between miRNAs and clinical parameters were analyzed using the chi-square test. The survival outcomes were compared using the log-rank test. The overall survival (OS) was measured from the date of diagnosis to the date of death due to any cause. The OS was censored at the date of the last follow-up visit. Two-sided tests were used in all calculations and *p* values < 0.05 were considered significant. Statistical analyses were performed with GraphPad Prism 5.0 (GraphPad Software, Inc., San Diego, CA) and the statistical software package IBM SPSS Statistics version 24.0 (IBM Corp., Armonk, NY).

## Results

### Upregulation of exosomal production during hypoxia

**Figure 1:**
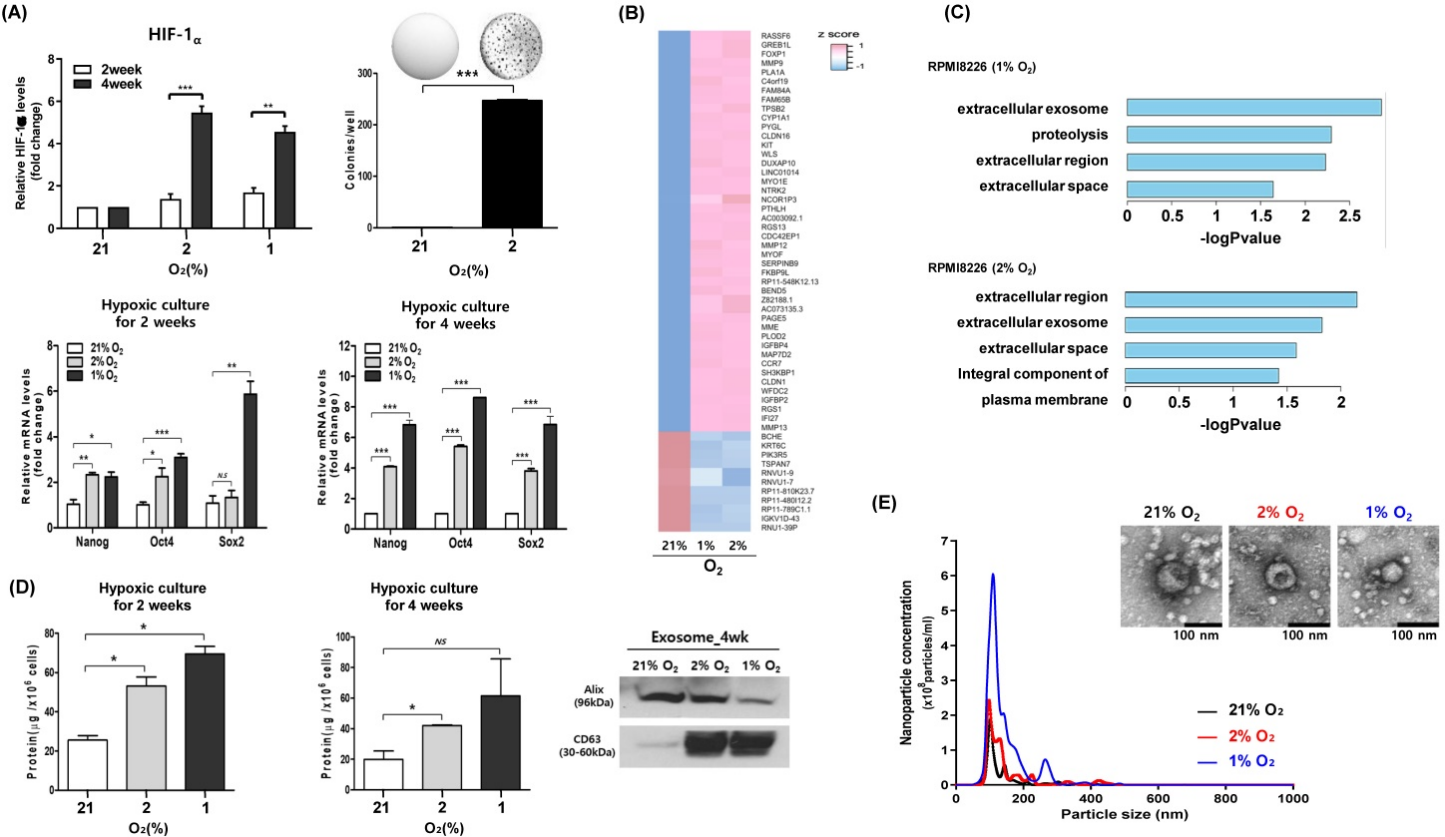
** Exosomal production is upregulated during long-term culture of myeloma cells under hypoxia.** RPMI 8226 cells were cultured under hypoxic conditions (1% or 2% O_2_) or normoxic conditions (21% O_2_) for the indicated times. (A) mRNA expression of HIF-1α and stem cell markers (Nanog, Oct4 and SOX2) were analyzed by RT-qPCR. Increased colony formation was found in hypoxic RPMI 8226 cells. The data represent the mean ± SEM of three independent experiments, *p < 0.05; **p < 0.01; ***p < 0.001. NS, not significant. (B) Heat map of the differentially expressed genes (DEGs) in both hypoxia conditions. (C) Gene ontology (GO) enrichment analysis for DEGs using DAVID. The bar graph shows GO term with the overlap ratio of more than 10% between DEGs and GO genes. (D) Protein concentration of exosomes derived from RPMI 8226 cells cultured under hypoxic or normoxic conditions for the indicated times. Western blot analysis of the exosomal markers Alix and CD63 from RPMI 8226 cells cultured for 4 weeks. (E) Representative particle concentration and size distribution of exosomes derived from RPMI 8226 cells cultured under hypoxic or normoxic conditions. Transmission electron microscopic images of exosomes (Scale bar: 100 nm).

We performed long-term hypoxic culture to mimic the hypoxic microenvironment of bone marrow. Thus, RPMI 8226 cells were cultured under hypoxic conditions (2% O_2_ and 1% O_2_) for 2 weeks and 4 weeks, and surviving myeloma cells were analyzed for hypoxia-induced phenotypic changes. When we compared the colony formation and expression of cell signaling pathways between myeloma cells cultured at hypoxic (2% O_2_) and normoxic conditions (21% O_2_), myeloma cells that survived under chronic hypoxia showed increased colony formation (Figure 1A). The expression of hypoxia-inducible factor (HIF)-1α was upregulated in myeloma cells under hypoxic conditions (2% O_2_ and 1% O_2_), and the upregulation of HIF-1α was more evident in myeloma cells cultured for 4 weeks compared with those cultured for 2 weeks (Figure 1A). Consistent with the association of hypoxia with stem cell-like characteristics, the expression of *Oct4, Sox2* and *Nanog* genes, which are known as embryonic stem cell markers, was increased in hypoxic conditions compared with normoxic conditions (Figure 1A). In transcriptome analysis, we compared changes between hypoxic and normoxic conditions. Fifty-five genes were differentially expressed between the two groups (Figure 1B). GO analysis confirmed that the genes detected in each group were related to the extracellular exosome (Figure 1C). Comparison of exosome production by hypoxic and normoxic myeloma cells showed there was increased production of exosomes from hypoxic myeloma cells (Figure 1D). Importantly, the size and morphology of exosomes produced by hypoxic myeloma cells were not different from those produced by parental cells at normoxic conditions (Figure 1E).

### Exosomal miRNA profiles of hypoxic myeloma cells

**Figure 2:**
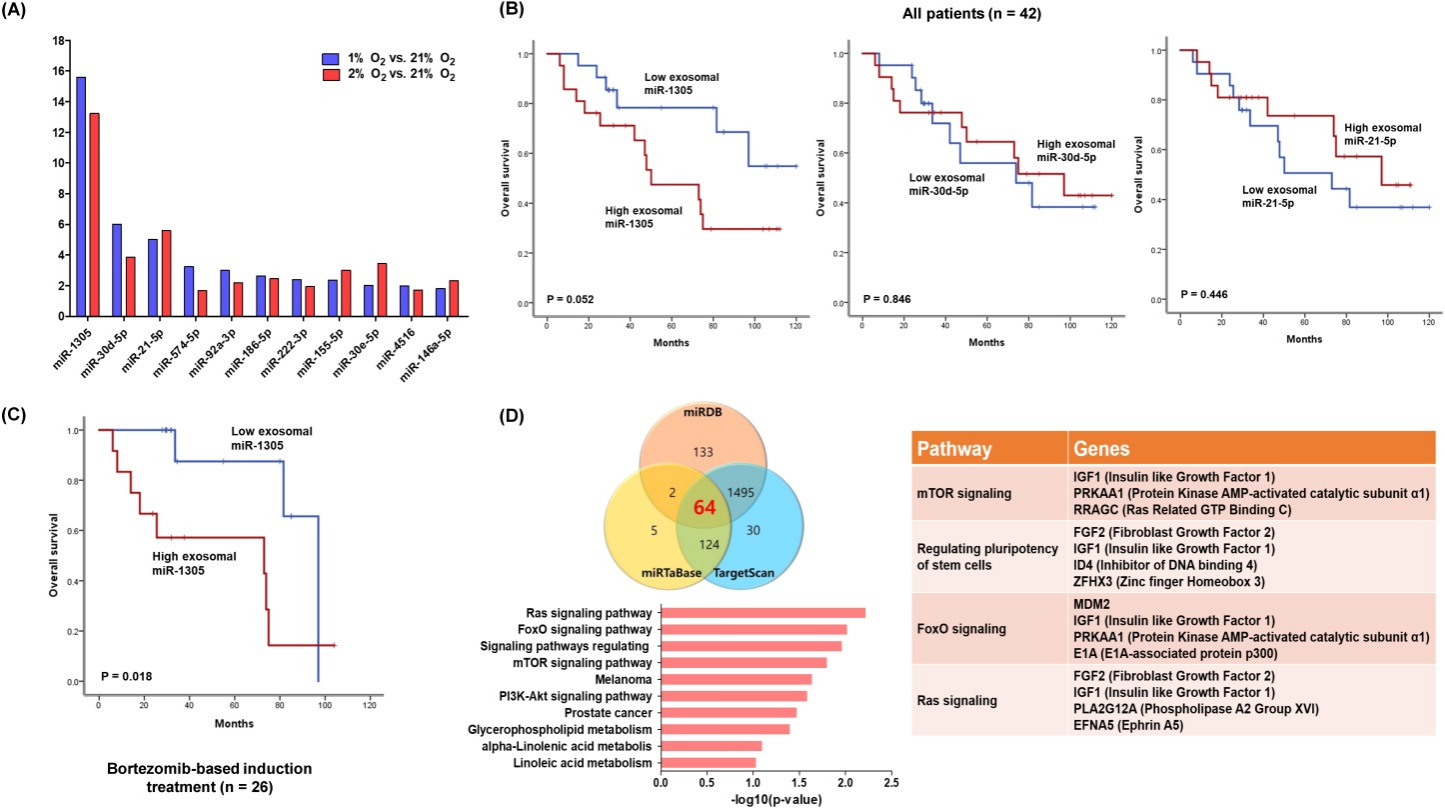
** miRNA profiles of hypoxic exosome and its association with overall survival rates in MM patients.** (A) Exosomal miRNA profiles of RPMI 8226 cells cultured under hypoxic conditions (1% or 2% O_2_). Fold change vs normoxic conditions (21% O_2_). (B) Kaplan-Meier curves representing the correlation between miRNA signature based on exosomal miR-1305, miR-30d-5p and miR-21-5p expression levels and overall survival rates in MM patients. (C) MM patients who were initially treated with bortezomib-containing chemotherapy showed a significantly poorer overall survival rate in the high exosomal miR-1305 group. (D) Target gene prediction of differentially expressed miR-1305. Venn diagram showing the overlap of miR-1305 target genes with a high rate of context score by using three different miRNA target analysis programs. Representative pathways of miR-1305 target genes are summarized.

We analyzed the expression of exosomal miRNAs from hypoxic and normoxic myeloma cells. Among the differentially expressed miRNAs, miR-1305, miR-30d-5p and miR-21-5p were in more than twofold greater abundance in the hypoxic exosomes compared with normoxic exosomes (Figure 2A). Based on the upregulated exosomal miRNA profiles of hypoxic myeloma cells, we analyzed the expression of miRNAs using archived serum samples of 42 myeloma patients. The clinical and laboratory characteristics of patients were summarized in Table 1. After diagnosis of myeloma, three induction regimens were used as follows: thalidomide-dexamethasone (n =16), bortezomib-thalidomide-dexamethasone (n = 11), and bortezomib-melphalan-prednisolone (n = 15). At the median follow-up of 85 months (95% Confidence Interval: 55.1-114.9 months), 19 deaths were confirmed. When we divided patients into high and low groups according to the median read counts of each exosomal miRNA, the group with high exosomal miR-1305 showed a tendency to poorer overall survival when compared with the low exosomal miR-1305 group (Figure 2B). However, exosomal miR-30d-5p and miR-21-5p failed to show any association with overall survival. The comparison of clinical and laboratory characteristics between high and low exosomal miR-1305 groups did not show any significant difference (Table 1). The subgroup analysis of overall survival in 26 patients who were initially treated with bortezomib-containing induction treatment showed a significantly worse overall survival by members of the high exosomal miR-1305 group compared with the low miR-1305 group (Figure 2C). We performed *in silico* analysis to determine possible miR-1305 targets using three database systems including miRDB, miRTaBase and TargetScan. Sixty-four transcripts with conserved miR-1305 binding sites were predicted. Predicted target genes were potentially involved in four major pathways (Ras signaling, FoxO signaling, regulation of stem cells and mTOR signaling) (Figure 2D, Supplementary Figure 1).

### High expression of miR-1305 target genes in hypoxic myeloma cells

**Figure 3:**
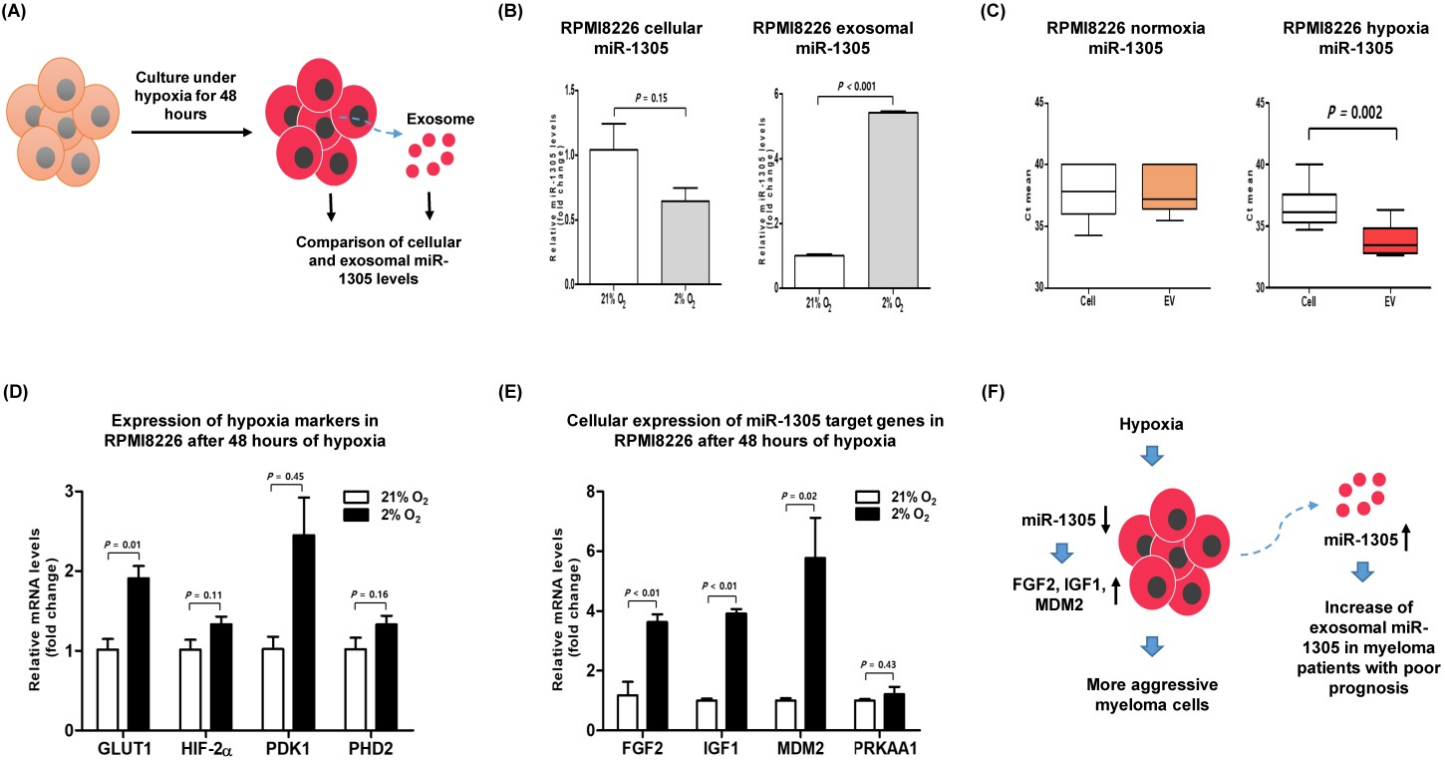
**Hypoxia-induced downregulation of cellular miR-1305 and upregulation of miR-1305 target genes in MM cells.** RPMI 8226 cells were cultured under normoxic conditions (21% O_2_) or hypoxic conditions (2% O_2_) for 48 h. (A) Schematic model for comparison of cellular and exosomal miR-1305 from RPMI 8226 cells cultured under hypoxia. (B) The level of cellular and exosomal miR-1305 was analyzed by RT-qPCR. Cellular miR-1305 level was decreased, but exosomal miR-1305 level was increased under hypoxia. (C) Cellular expression of hypoxia markers (GLUT1, HIF2α, PDK1 and PHD2) was upregulated in RPMI 8226 cells cultured under hypoxia. (D) Cellular expression of the miR-1305 candidate targets (FGF2, IGF1 and MDM2) in RPMI 8226 cells cultured under hypoxia. (E) Schematic model for hypothetical consequences caused by myeloma cells cultured under hypoxia. Upregulation of miR-1305 target gene expression was caused by reduced cellular miR-1305 in hypoxic RPMI 8226 cells, which may lead to more aggressive myeloma cells and myeloma patients with poor prognosis.

To validate the role of miR-1305 in myeloma cells during hypoxia, we compared the levels of cellular and exosomal miR-1305 in RPMI 8226 cells after 48 h of culture under hypoxic conditions (Figure 3A). The level of cellular miR-1305 decreased whereas exosomal miR-1305 increased during hypoxia (Figure 3B). However, the level of cellular miR-1305 was not different from exosomal miR-1305 levels under normoxic conditions (Figure 3C). An increase of hypoxia-related markers, including GLUT1, HIF2-α, PDK1 and PHD2 was observed, consistent with long-term hypoxia for 2 and 4 weeks (Figure 3D). Under hypoxic conditions, the cellular expression of miR-1305 targets, including *FGF2, IGF1* and *MDM2* was upregulated compared with that under normoxic conditions (Figure 3E). This inverse association of cellular miR-1305 with target genes was also observed in miR-1305 transfected RPMI 8226 cells (Supplementary Figure 2). Considering that cellular miR-1305 was downregulated under hypoxia, the upregulation of cellular miR-01305 target gene expression - under hypoxia might be caused by the export of miR-1305 via exosomes (Figure 3F).

### Effects of exosomal miR-1305 on macrophages

**Figure 4:**
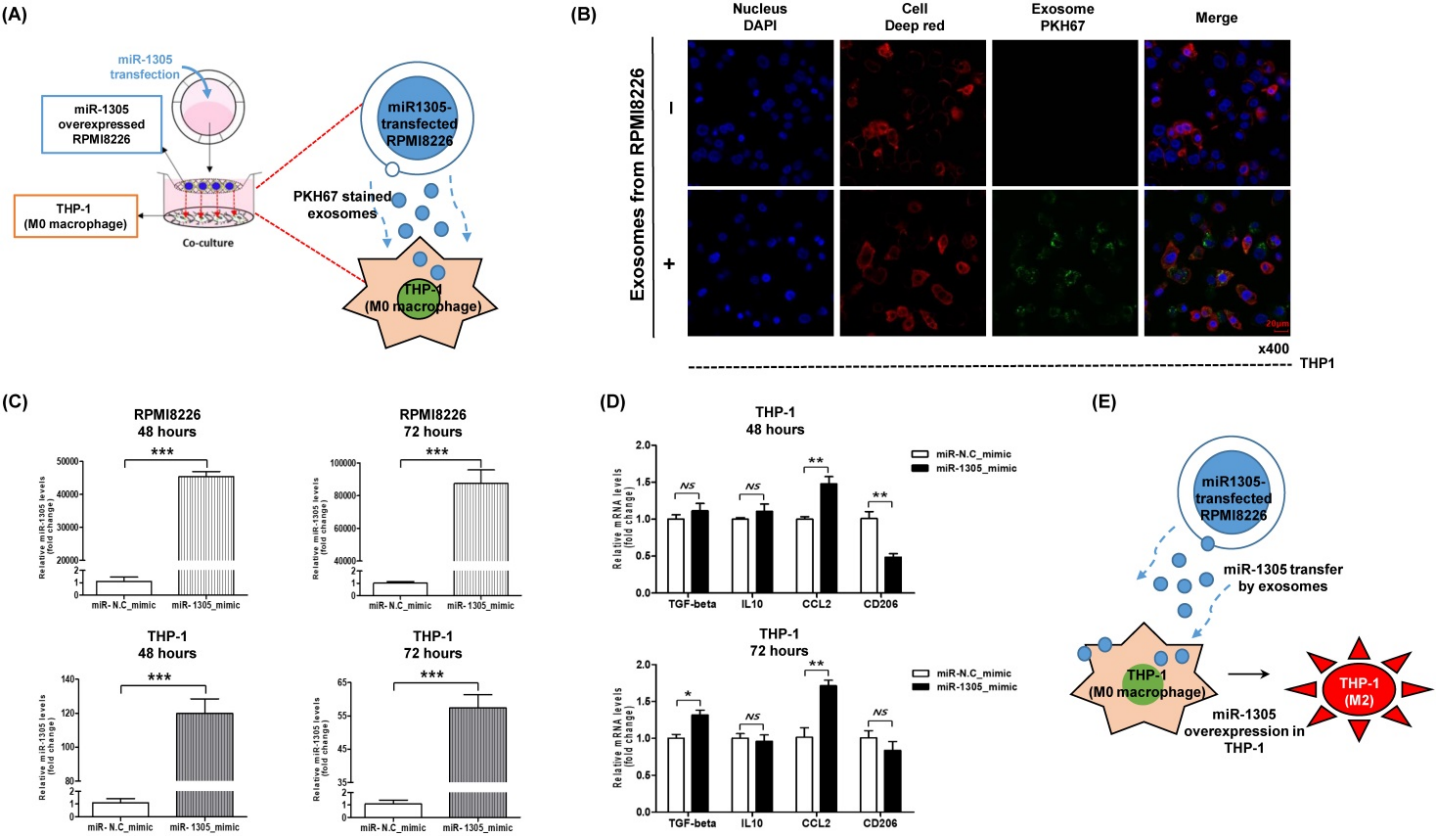
** Transfer of miR-135b derived from myeloma cells to THP-1 macrophages via exosomes.** (A) Schematic model of the experiment to confirm the effects of exosomal miR-1305 on the tumor microenvironment. THP-1 macrophage cells were seeded in the lower chamber of a Transwell plate, while miR-1305-transfected RPMI 8226 myeloma cells were seeded on the top of the insert. (B) Internalization of myeloma cell-derived exosomes by THP-1 cells. THP-1 macrophage cells were cultured with PKH67-labeled exosomes derived from RPMI 8226 cells. Representative confocal microscopy image shows the PKH67-labeled exosome (green) signals detected in the cytoplasm of THP-1 macrophage cells (red). Nuclear counterstaining was performed using DAPI (blue). (C) Quantitative RT-PCR for miR-1305 was carried out using RNA isolated from RPMI 8226 and THP-1 cells after 48 h and 72 h of coculture. The data represent the mean ± SEM of three independent experiments. ***p < 0.001. (D) Expression of M2 macrophage markers in THP-1 cells in response to exosomes derived from RPMI 8226/miR-1305_mimic or control (RPMI 8226/miR-NC_mimic). Quantitative RT-PCR for TGF-β, IL10, CCL2 and CD206 was carried out using RNA isolated from THP-1 cells after 48 h or 72 h of coculture. TGF-β and CCL2 expression was upregulated by the addition of RPMI 8226/miR-1305_mimic exosomes compared with control. (E) Proposed model in this study for how myeloma-derived miR-1305 may promote exosome-induced macrophage polarization in multiple myeloma.

To explore the effects of exosomal miR-1305 derived from myeloma cells on immune cells of the tumor microenvironment, we cocultured miR-1305-transfected RPMI 8226 cells and THP-1 macrophage cells (Figure 4A). After coculture, we confirmed the movement of RPMI 8226-derived exosomes into THP-1 cells (Figure 4B). Accordingly, intracellular miR-1305 levels increased in RPMI 8226 as well as in THP-1 cells after 48 h and 72 h of coculture (Figure 4C). Then we analyzed the expression of M2 macrophage markers in THP-1 cells after 48 h and 72 h of coculture with miR-1305-transfected RPMI 8226 cells. Coculture with miR-1305-transfected MM cells induced expression of TGF-β and CCL2 in THP-1 cells (Figure 4D). These results suggest that exosomal miR-1305 may be responsible, at least in part, for the M2 polarization of THP-1 promoting the pro-tumor effects of macrophages (Figure 4E).

## Discussion

Exosomes have been reported to play a role in the tumorigenesis of many cancer types, particularly through transfer of miRNA silencing of messenger RNA in target cells 32. Although cell-free miRNAs exist in blood, exosomal miRNAs could reflect the biology of tumor cells more exactly because tumor cells secrete exosomes containing miRNAs by active processes, whereas cell-free miRNAs are released from dead tumor cells by apoptosis or necrosis 33. This study analyzed the effect of hypoxic MM cell-derived exosomal miRNAs on tumor aggressiveness and its association with poor outcomes in MM patients. Our hypoxia model showed increased expression of stem cell markers in hypoxic MM cells compared with normoxic parental cells, and the overexpression of exosome production-related genes was found in the profiling of differentially expressed genes in hypoxic MM cells. Accordingly, the number of exosomes produced from hypoxic MM cells was significantly higher than that from normoxic parental cells. Our results are consistent with the previous study reporting a twofold increase of exosomes secreted from hypoxia-resistant MM cells cultured at 1% O_2_, mimicking *in vivo* conditions of hypoxic BM 34.

Our comparison of exosomal miRNAs between hypoxic and normoxic MM cells identified differentially expressed exosomal miRNA profiles of hypoxic MM cells. Thus, the expression of miR-1305 was significantly higher in exosomes isolated from hypoxic MM cells at 1% and 2% O_2_ than those from normoxic parental MM cells (Figure 2A). Although a previous study reported the prognostic relevance of exosomal let-7b and miR-18a for the first time in MM patients 35, exosomal miR-1305 has never been reported in MM cells. Indeed, the tumor-suppressive role of miR-1305 was recently suggested in non-small cell lung cancer (NSCLC) tissues and cell lines 36. Thus, miR-1305 inhibited the proliferation of NSCLC cells, and *MDM2* was proven as a target of miR-1305, consistent with our study. Furthermore, miR-1305 was reported to inhibit the stem cell properties of liver cancer stem cells in human hepatocellular carcinoma 37. Thus, miR-1305 disrupted the activation of the AKT-signaling pathway and repressed colony formation and tumorigenicity of cancer stem cells. Our study showed the overall survival of patients with high exosomal miR-1305 was poorer than patients with low exosomal miR-1305. This survival difference was more prominent in patients receiving bortezomib-based induction treatment, even though other exosomal miRNAs such as miR-30d-5p and miR-21-5p failed to show a significant association with overall survival (Figure 2B, C). The exploration of miR-1305 targets using the database (miRDB, miRTaBase and TargetScan) for target genes of miRNAs, revealed well-known genes essential for myeloma cell growth and proliferation such as insulin-like growth factor type 1 (IGF-1) and mouse double minute 2 homolog (MDM2) 38, 39. Furthermore, miR-1305 can target genes regulating pluripotency of stem cells such as fibroblast growth factor 2 (FGF2) which, out of 22 members of the human FGF family, was uniquely expressed in the majority of MM cell lines 40. Instead, miR-1305 was reported to have a regulatory role in the pluripotency of human embryonic stem cells because overexpression of miR-1305 induced stem cell differentiation 41.

Our study found an increase of miR-1305 in exosomes and a decrease of cellular miR-1305 under hypoxia even though there was no difference between the cellular and exosomal levels of miR-1305 under normoxic conditions (Figure 3B, C). Accordingly, increased expression of cellular *IGF1, MDM2* and *FGF2*, all targets of miR-1305, was observed under hypoxic compared with normoxic conditions (Figure 3E). These findings imply that hypoxia decreases cellular levels of miR-1305 via the exosome-mediated export of miR-1305, leading to increased expression of cellular *IGF1, MDM2* and *FGF2*. Consistent with a pro-tumor effect of exosome-mediated export of tumor-suppressive miR-1305, the aforementioned study also showed that patients with high exosomal let-7 had better survival rates than patients with low exosomal let-7 35. Because let-7 acting as a tumor suppressor could inhibit oncogenes such as *CCND1, MYC* and *RAS*, decrease of cellular let-7 may lead to cell proliferation 35, 42. Indeed, exosome-mediated secretion of tumor-suppressor miRNA has been reported as a mechanism to coordinate activation of metastasis as well as cellular disposal of tumor-suppressor miRNAs in other cancer types 43-45. Thus, hypoxic MM cells could maintain their growth and cancer stem cell-like characteristics via exosome-mediated disposal of cellular miR-1305 under hypoxia (Figure 3F). Cell-to-cell communication between MM cells and BM stromal cells is another important function of exosomal miRNAs because they enhance tumor aggressiveness, including angiogenesis 46. A previous study demonstrated that exosomal miR-135b released from hypoxia-resistant MM cells suppressed target endothelial cells, leading to enhanced endothelial tube formation under hypoxia 34. The tumor microenvironment comprises fibroblasts, stromal cells and immune cells such as macrophages. In our study, miR-1305 was transferred from MM cells to macrophages and miR-1305-transferred macrophages showed tumor-promoting M2 macrophage phenotypes (Figure 4B, C, D). These results suggest that another function of exosomal miR-1305 is to influence adjacent immune cells of the tumor microenvironment (Figure 4E).

In conclusion, we demonstrated that exosome-mediated secretion of miR-1305 in MM cells under hypoxia promoted tumor aggressiveness. We also found unfavorable survival outcomes in myeloma patients with high circulating exosomal miR-1305. Considering that exosomal miRNAs may represent the biology of myeloma more accurately than cell-free miRNAs, the assessment of exosomal miR-1305 may be useful for predicting disease outcome and for establishing a risk-adapted treatment approach for myeloma patients. Further studies are required to fully elucidate the underlying mechanisms of the function of exosomal miR-1305 and its use as a biomarker in the clinic.

## Supplementary Material

Supplementary figures and tables.

## Figures and Tables

**Table 1:** Characteristics of patients

Characteristics	ExosomalmiR-1305		*P* value
	Low (n = 21)	High (n = 21)	
**Age**			
< 60	11 (52)	9 (43)	0.758
≥ 60	10 (48)	12 (57)	
**Sex**			
Male	10 (48)	10 (48)	> 0.999
Female	11 (52)	11 (52)	
**ISS**			
Low	7 (33)	10 (48)	0.580
Intermediate	9 (43)	8 (38)	
High	5 (24)	3 (14)	
**Serum LD**			
Normal	9 (43)	15 (71)	0.137
Increased	11 (52)	6 (29)	
Unknown	1 (5)	0 (0)	
**Bone marrow plasma cell**			
< 30%	8 (38)	8 (38)	> 0.999
≥ 30%	13 (62)	13 (62)	
**Deletion 13**			
Absence	9 (43)	16 (76)	0.076
Presence	9 (43)	3 (14)	
Unknown	3 (14)	2 (10)	
**Deletion 17p**			
Absence	17 (81)	19 (90)	0.519
Presence	1 (5)		
Unknown	3 (14)	2 (10)	
**Translocation (4;14)**			
Absence	14 (67)	18 (86)	0.287
Presence	4 (19)	1 (5)	
Unknown	3 (14)	2(10)	
**Translocation (11;14)**			
Absence	9 (43)	11 (52)	0.549
Presence	3 (14)	1 (5)	
Unknown	9 (43)	9 (43)	
**Translocation (14;16)**			
Absence	18 (86)	19 (90)	> 0.999
Presence	0 (0)	0 (0)	
Unknown	3 (14)	2 (10)	
**Translocation (14;20)**			
Absence	13 (62)	10 (48)	0.536
Presence	0 (0)	0 (0)	
Unknown	8 (38)	11 (52)	
**First-line induction treatment**			
Bortezomib-based treatment	14 (67)	12 (57)	0.751
Thalidomide-based treatment	7 (33)	9 (43)	
**Autologous stem cell transplantation**			
Done	15 (71)	11 (52)	0.341
Not done	6 (29)	10 (48)	
**Survival status**			
Alive	15 (71)	8 (38)	0.062
Dead	6 (29)	13 (62)	
