# Supplementary material for: Exosomal miR-1305 in the oncogenic activity of hypoxic multiple myeloma cells: a biomarker for predicting prognosis
Source: J Cancer. 2021 Mar 14;12(10):2825–34. doi: 10.7150/jca.55553 (PMC8040895; doi:10.7150/jca.55553)
Supplement: Supplementary file 1 — Supplementary figures and tables. [file jcav12p2825s1.pdf]

Supplementary figure 1

Entrez ID	Target gene	Description
2247	FGF2	Fibroblast growth factor 2
3479	IGF1	Insulin like growth factor 1
4193	MDM2	MDM2 proto-oncogene
5562	PRKAA1	Protein kinase AMP-activated catalytic subunit alpha 1
160418	TMTC3	Transmembrane and tetratricopeptide repeat containing 3
90007	MIDN	Midnolin
2005	ELK4	ELK4, ETS transcription factor
3400	ID4	Inhibitor of DNA binding 4, HLH protein
23554	TSPAN12	Tetraspanin 12
51014	TMED7	Transmembrane p24 trafficking protein 7
84255	SLC37A3	Solute carrier family 37 member 3
182	JAG1	Jagged 1
7750	ZMYM2	Zinc finger MYM-type containing 2
3157	HMGCS1	3-hydroxy-3-methylglutaryl-CoA synthase 1
5775	PTPN4	Protein tyrosine phosphatase, non-receptor type 4
23401	FRAT2	FRAT2, WNT signaling pathway regulator
149628	PYHIN1	Pyrin and HIN domain family member 1
84437	MSANTD4	Myb/SANT DNA binding domain containing 4 with coiled-coils
440026	TMEM41B	Transmembrane protein 41B
10140	TOB1	Transducer of ERBB2, 1
64376	IKZF5	IKAROS family zinc finger 5
55917	CTTNBP2NL	CTTNBP2 N-terminal like
463	ZFHX3	Zinc finger homeobox 3
130507	UBR3	Ubiquitin protein ligase E3 component n-recognin 3
154043	CNKSR3	CNKSR family member 3
116064	LRRC58	Leucine rich repeat containing 58
29982	NRBF2	Nuclear receptor binding factor 2
10095	ARPC1B	Actin related protein 2/3 complex subunit 1B
7543	ZFX	Zinc finger protein X-linked
30011	SH3KBP1	SH3 domain containing kinase binding protein 1
83857	TMTC1	Transmembrane and tetratricopeptide repeat containing 1
84553	FAXC	Failed axon connections homolog
54964	C1orf56	Chromosome 1 open reading frame 56
9060	PAPSS2	3'-phosphoadenosine 5'-phosphosulfate synthase 2
1747	DLX3	Distal-less homeobox 3
80816	ASXL3	ASXL transcriptional regulator 3
90624	LYRM7	LYR motif containing 7
56902	PNO1	Partner of NOB1 homolog
116	ADCYAP1	Adenylate cyclase activating polypeptide 1
27345	KCNMB4	Potassium calcium-activated channel subfamily M regulatory beta subunit 4
54891	INO80D	INO80 complex subunit D
80312	TET1	Tet methylcytosine dioxygenase 1
7678	ZNF124	Zinc finger protein 124
11145	PLA2G16	Phospholipase A2 group XVI
7782	SLC30A4	Solute carrier family 30 member 4
29957	SLC25A24	Solute carrier family 25 member 24
8975	USP13	Ubiquitin specific peptidase 13
55161	TMEM33	Transmembrane protein 33
64121	RRAGC	Ras related GTP binding C
1946	EFNA5	Ephrin A5
160851	DGKH	Diacylglycerol kinase eta
79589	RNF128	Ring finger protein 128, E3 ubiquitin protein ligase
54715	RBFOX1	RNA binding fox-1 homolog 1
85463	ZC3H12C	Zinc finger CCCH-type containing 12C
7328	UBE2H	Ubiquitin conjugating enzyme E2 H
7071	KLF10	Kruppel like factor 10
54800	KLHL24	Kelch like family member 24
79628	SH3TC2	SH3 domain and tetratricopeptide repeats 2
10345	TRDN	Triadin
10787	NCKAP1	NCK associated protein 1
90321	ZNF766	Zinc finger protein 766
81579	PLA2G12A	Phospholipase A2 group XIIA
5504	PPP1R2	Protein phosphatase 1 regulatory inhibitor subunit 2
2033	EP300	E1A binding protein p300

Supplementary figure 2

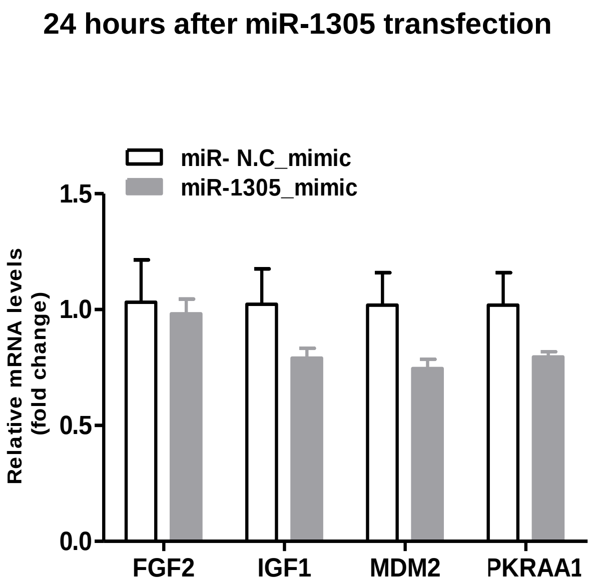

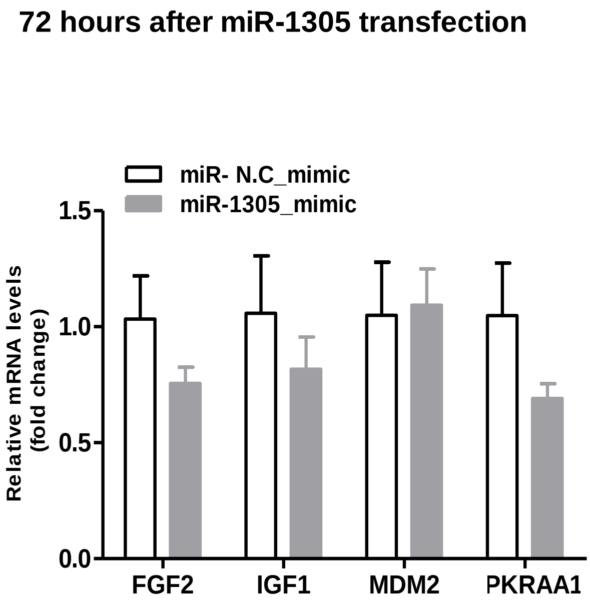

Cellular expression of FGF2, IGF1, MDM2 and PKRAA1 was changed in miR-1305 transfected RPMI8226.
